# Community Malaria Knowledge, Experiences, Perceived Roles, and Acceptability of Community-Directed Distribution of Intermittent Preventive Therapy for Pregnancy in Rural Southeast Nigeria

**DOI:** 10.1155/2022/8418917

**Published:** 2022-01-18

**Authors:** Ijeoma Nkem Okedo-Alex, Ifeyinwa Chizoba Akamike, Johnbosco Ifunanya Nwafor, Chihurumnanya Alo, Adaoha Pearl Agu, Dejene Derseh Abateneh, Chigozie Jesse Uneke

**Affiliations:** ^1^African Institute for Health Policy and Health Systems, Ebonyi State University, Abakaliki, Nigeria; ^2^Department of Community Medicine, Alex Ekwueme Federal University Teaching Hospital Abakaliki Ebonyi State, Nigeria; ^3^Department of Obstetrics and Gynaecology, Alex Ekwueme Federal University Teaching Hospital, Abakaliki, Ebonyi State, Nigeria; ^4^Kotebe Metropolitan University, Menelik II College of Medicine and Health Sciences, Department of Medical Laboratory Sciences, Addis Ababa, Ethiopia

## Abstract

**Background:**

The community plays key roles in protecting pregnant women in rural areas from malaria. This study assessed malaria experiences, knowledge, perceived roles in malaria prevention in pregnancy, and acceptability of community-directed distribution of intermittent preventive therapy (IPTp) for malaria in pregnancy in rural Southeast Nigeria.

**Methods:**

This study presents part of the baseline findings of a before-and-after study. Data was collected from 817 community members in Ebonyi State using interviewer-administered questionnaires and focus group discussions (FGDs). Data were analyzed using SPSS version 20 and thematic analysis.

**Results:**

The majority of the respondents were females (73.8%) with a mean age of 36.08 ± 15.4. Most respondents (65.2%) had Insecticide-Treated Net (ITN) and fever in the past year (67.1%). Malaria (88.6%) was identified as the major health condition in the community. Majority (74.1%) knew infected mosquito bites as the cause of malaria while 61.1% and 71.5% were definitely sure that pregnant women and children were at risk for malaria. Sleeping under ITN (54.3%), clean environment (39.7%), and herbal medications (26.8%) were the main ways of malaria prevention cited. Only 18.4% of the participants rated their knowledge of IPTp as adequate, and only 9.3% knew the common drug names used for IPTp. The major perceived roles in malaria prevention in pregnancy were referral of pregnant women to the health facility, encouragement of household ITN use, and sustaining malaria-related projects. The majority of the participants (60.6%) strongly agreed that community-directed distribution of IPTp-SP will improve the prevention of malaria in pregnancy. Most (77.2%) considered community-directed distribution of IPTp acceptable, and 74.4% of the pregnant respondents preferred community to facility administration of IPTp.

**Conclusions:**

Malaria was recognized as a prevalent disease, but there was inadequate knowledge of malaria prevention in pregnancy notably intermittent preventive therapy. There was positive perception of roles in malaria prevention in pregnancy and high acceptability of community-directed distribution of IPTp. Community-level malaria control programs should utilize a whole-of–community approach to optimally engage and educate the community on malaria prevention in pregnancy as well as explore community distribution approach for IPTp.

## 1. Introduction

According to the World Malaria Report 2021 (page xv), the World Health Organization (WHO) African Region continues to bear the global malaria burden, accounting for about 95% of the 241 million malaria cases worldwide and 96% of the 627000 deaths from malaria in 2020 [1]. Nigeria significantly contributes to the global burden of malaria cases as 27% of all malaria cases in 2020 occurred in Nigeria [1]. Malaria is holoendemic in Nigeria and remains a major public health problem, taking its greatest toll on children under the age of 5 and pregnant women, although it is preventable, treatable, and curable [2]. Malaria in pregnancy (MIP) is associated with varying symptoms and can be complicated with anemia, stillbirths, prematurity, and low birth weight (LBW). Malaria-associated maternal illness and low birth weight are mostly the result of *Plasmodium falciparum* infection and occur frequently in Nigeria [3]. The disease overstretches the nation's struggling health system with grave socio-economic consequences for both household and national levels. This is manifested in reduced gross domestic product, productivity, and high out-of-pocket expenditures on treatment and prevention expenditures [4].

The transmission of malaria in Nigeria is endemic and perennial. There are favorable climatic conditions and a higher prevalence of malaria after the end of the rainy season [5, 6]. The predominant Anopheline vector species in Nigeria are *An. gambaie sp*, *An. arabiensis*, and *An. Funestus*. Malaria transmission is highest in rural communities with enabling environmental conditions for vector breeding, and this is further compounded by the fragile health system arrangements in these areas. Pregnant women and under-five children in such remote areas remain worse hit by malaria [7].

To prevent and ensure proper treatment of malaria in pregnancy, the three cardinal approaches recommended by the World Health Organization (WHO) are the use of long-lasting Insecticide-Treated Nets (ITNs), prompt diagnosis, and effective treatment of malaria infection and intermittent preventive treatment in pregnancy with suphadoxine-pyrimethamine (IPTp-SP) as part of antenatal services in areas of moderate to high transmission of *P. falciparum* [3].

At the community and household level, effective prevention and control of malaria are largely dependent on community members properly identifying malaria as a priority health issue. The community's knowledge and perceptions of malaria could influence practices that predispose or protect from malaria [8, 9].

The community could play key roles in protecting pregnant women from malaria by encouraging communal practices and norms such as spousal involvement, community support of antenatal attendance, ITN and IPTp use, and environmental sanitation as well as collaborating with and sustaining health programs on malaria prevention. In 2018, only 46% of Nigerian women in rural areas had at least four antenatal care (ANC) visits, and only 14% of ANC users initiated these visits in the first trimester [6]. This conspicuously falls short of the minimum eight visits and early ANC initiation advocated by the WHO [10]. Although Nigeria reflected the WHO recommendations of eight contact visits in its 2017 ANC orientation package for health care providers [11], this most recent national survey on ANC utilization was based on attending at least four ANC visits. Suboptimal antenatal care utilization and by extension, IPTp uptake remains a burgeoning concern in the prevention of malaria in pregnancy in Nigeria [6].

Community approaches have been increasingly recognized because they improve community acceptability and ownership as the structures used are part of the community in addition to the existing community solidarity. They also obviate the creation of new structures for health interventions [12, 13]. Studies have shown that community-based programs can substantially increase effective access to IPTp and other types of malaria prevention in addition to increasing access to antenatal care and other formal health care access in general [14–16]. Such community-level programs include community-directed distribution of preventive malaria commodities such as IPTp-SP by trained community distributors, community sensitization, and community case management of malaria by community health workers [14, 17, 18]. Although such community-level interventions have been shown to be contextually relevant and effective in resource-constrained settings [14, 19, 20], very few studies have assessed their acceptability by community members as acceptability can be an important precursor of sustainability. The aim of this study was to assess community malaria experiences, knowledge, perceived roles in prevention of malaria in pregnancy, and acceptability of community-directed distribution of intermittent preventive therapy for malaria in pregnancy in rural Southeast Nigeria. The findings are part of the baseline findings of an intervention study to implement community-directed distribution of intermittent preventive therapy for the prevention of malaria in pregnancy [19].

## 2. Materials and Methods

### 2.1. Study Area

This study was conducted in three communities (Okuzzu-Ukawu, Isinkwo, and Abomege) located in Ukaba Development Centre of Onicha local government area (LGA) in Ebonyi State, Southeast Nigeria. Ebonyi State has three senatorial zones and thirteen LGAs. According to the 2006 population and housing census, the population of Ebonyi state is approximately 2,176,947 with a landmass of 5,935 square kilometers. Infants (children < 1 year old) make up 4% of the population, children under five 20%, and women of childbearing age 22% of the population [21]. The people of Ukawu are mostly Ibos, the dominant tribe of southeast geopolitical zone of Nigeria, and their major occupations include farming and trading.

Using the most recent national census (1991) definition, a rural area is defined as a settlement with <20000 inhabitants [22]. The study area had <20000 inhabitants. Nigeria has also been shown to be dominantly rural [23].

Ebiriogu community located in Okuzzu-Ukwau political ward was the intervention community for the community-directed IPTp distribution project. More details about the intervention have been published elsewhere [19]. The community has three settlements and one primary health center (PHC) which is the major source of orthodox health care services in the community. However, people of the community also access health services in the PHCs located in the other political wards as well as from traditional healers.

### 2.2. Study Population

Data was collected from adult men and women in the settlements in Ebirogu community. Only those who resided in the community and gave informed consent were surveyed. To ensure adequate participation of women of childbearing age who are the primary targets of IPTp, nursing and pregnant mothers in the community were encouraged to participate in the study. Also, those attending immunization and antenatal clinics in the high patronage PHCs in Ukawu Development center were consecutively recruited into the study (for the interviews) over a three-week period. Opinion leaders and pregnant/nursing mothers in the community participated in the focus group discussions.

### 2.3. Study Design

The study utilized a descriptive cross-sectional study design using a mixed method approach comprising semistructured interviews and focus group discussions.

### 2.4. Sample Size Determination and Sampling

The WINPEPI statistical software for epidemiologists was used to calculate the minimum sample size using the formula for a single proportion [24, 25]. We assumed that at least 50% of the respondents would consider community-directed distribution of IPTp acceptable. A minimum sample size of 385 was calculated and recalculated to 427 after adjusting for a nonresponse rate of 10%. To improve the robustness (representativeness and precision) of the study, the sample size was doubled to 854. Eight hundred and seventeen participants responded to the questionnaires to give a response rate of 95.7%. Using the settlements within the community as clusters, two clusters were selected by balloting and all households in each cluster sampled for eligible participants in the community. All eligible participants who provided written informed consent were surveyed.

### 2.5. Data Collection Methods

Semistructured interviewer-administered questionnaires and focus group discussions (FGD) were used for data collection. Data was collected before the onset of the community-directed distribution of IPTp as part of the baseline data in May-June 2019. The questionnaire had three sections, and the first section collected information on socio-demographic and malaria-related characteristics, burden of malaria and knowledge of prevention using IPTp and community ownership, and acceptability of community-directed distribution of IPTp. The second section explored the burden of malaria, knowledge of the causes, at-risk-populations, and intermittent preventive therapy for malaria in pregnancy. The third section of the questionnaire explored perceptions on community roles, ownership, and acceptability of community-directed distribution of IPTp for prevention of malaria in pregnancy. The questionnaire was adapted from domains on malaria explored in the National Malaria Indicator Survey [2]. It was pretested among adult community members in another rural community who are not part of the study population in order to improve the clarity and arrangement of the questions. The questionnaires were administered by trained graduate research assistants.

The FGDs were used to further explore and understand community perceptions on the prevalent health challenges and burden of malaria in the community, community roles in preventing malaria in pregnancy, and acceptability of community-directed distribution of IPTp for prevention of malaria in pregnancy.

Two FGDs were held using a focus group discussion guide developed by the researchers. The FGD guide had a total of eight questions. Each FGD had 10-12 participants and lasted for about 40 minutes. One of the FGDs was held with nursing/pregnant mothers while the other FGD was held with opinion leaders in the community. With the assistance of the officer-in-charge of the PHC in Ebiriogu who acted as a link person, we purposively selected male and female opinion leaders because the project is aimed at ascertaining major health issues in the community and community support towards the project. They were encouraged to air their views freely because adequate understanding of the subject under study required that participants freely articulate and vocalize their views. The participants were approached face-to-face, and the purpose of the research was clearly explained to them. They were at liberty to opt out at will, but all agreed to participate. We used an electronic recorder to record the discussions and obtained permission to record from the respondents before commencing the FGDs. The officer-in-charge of the PHC in Ebiriogu also facilitated the selection of nursing and pregnant mothers in the community on the basis of willingness to participate in the FGD.

The principal researcher (female) moderated the discussions, and she was assisted by a research assistant who acted as the note-taker to record the significant inputs made by the discussants including nonverbal reactions. To ensure that the opinions of the FGD participants were appropriately captured, the moderator intermittently summarized the responses and sought their feedbacks on the accuracy of the summaries.

### 2.6. Data Management

#### 2.6.1. Measurement of Variables

The questionnaire was used to collect data on the independent and dependent variables. The socio-demographic and malaria-related characteristics of the respondents such as age, gender, marital status, occupation, experience of fever, and malaria were the independent variables.

The dependent variables were as follows:

- *Health Challenges and Burden of Malaria in the Community*. A multichoice question explored the three major health challenges in the community. A 5-point Likert scale question also assessed whether malaria was the most common disease in the community. This was scored as 1 point = definitely no, 2 points = probably no, 3 points = unsure, 4 points = probably yes, and 5 points = definably yes
- *Knowledge of Malaria and Malaria Prevention in Pregnancy*. A total of 11 questions were used to evaluate knowledge of malaria and malaria prevention in pregnancy. Seven of the questions assessed knowledge of at-risk groups and prevention of malaria in pregnancy. They were measured on a 5-point Likert scale scored as 1 point = definitely no, 2 points = probably no, 3 points = unsure, 4 points = probably yes, and 5 points = definably yes. One of the questions was on self-rating of the adequacy of the knowledge of intermittent preventive therapy in pregnancy. Three multichoice questions assessed the cause of malaria, measures for malaria prevention in pregnancy, and the recommended drug for IPTp
- *Perceived Roles and Community Ownership of Prevention of Malaria in Pregnancy*. Eight questions were used to measure this variable. Seven questions assessed the roles of the community in preventing malaria in pregnancy, while one question was on the participation of the community in health programs
- *Acceptability of Community-Directed Distribution of IPTp*. Three questions measured the acceptability, importance of, and willingness to support community-directed distribution of IPTp. A single question explored the preferred route of administration (facility vs. community) of IPTp-SP among pregnant women only

Data was checked, validated, and stored in a password-enabled computer only accessible to the primary researcher.

### 2.7. Data Analysis

#### 2.7.1. Quantitative Data Analysis

The Statistical Package for Social Sciences (SPSS) for Microsoft Windows version 20 software [26] was used for data analysis. We calculated frequencies and proportions for categorical variables while mean and standard deviations were calculated for numeric variables.

#### 2.7.2. Qualitative Data Analysis

Thematic analysis was used for qualitative data analysis. First, we transcribed the audio recordings from the focus group discussions and compared the transcripts with the handwritten notes in order to ensure that no important data was missed. We included some verbatim responses that reflected the original ideas of the participants. We then developed the preliminary coding framework from themes ideated from the discussion guide. The final coding framework was arrived at by comparing the transcripts with the preliminary coding framework in order to detect other themes. The data coding was done by two data coders, and disagreements were resolved using discussions and consensus. Following this, we then applied the final coding framework all the transcripts. The following themes were in the final coding framework: (a) major health problems in the community; (b) general knowledge of malaria, malaria in pregnancy, and prevention; (c) acceptability of community-directed distribution of IPTp; (d) perceived potential challenges of community-directed distribution of IPTp; and (e) sustainability of community-directed distribution of IPTp.

### 2.8. Ethics Approval

Ethical approval for this study was obtained from the Research and Ethics Committees of Ebonyi State Ministry of Health and the Ebonyi State University, Ebonyi State of Nigeria. No approval number (s) was issued. Written informed consent was obtained from the respondents.

## 3. Results

### 3.1. Quantitative Results

The mean age of the respondents was 36.08 ± 15.4. The majority of the respondents were females (73.8%), married (75.4%), and with 49.1% having up to secondary education (Table 1).

Most of the respondents had long-lasting ITN (65.2%) and were definitely sure of ever having had fever in the past one year (67.1%), and over four-fifths had fever diagnosed as malaria (81.4%). Over half of those with fever were treated with antimalarial from a health facility (58.2%) (Table 2).

Infected mosquito bites were identified as the cause of malaria by 74.1% of the participants. Only 10.8% and 7.6% of the participants rated their knowledge of IPTp as very adequate and adequate, respectively. Most participants did not know the name of the drug used for IPTp (83.5%). Only 7.2% and 2.1% knew that Fansidar and Amalar, respectively (common brands of *sulfadoxine-pyrimethamine*) were used for IPTp. Sleeping under treated mosquito treated net (54.3%) was the most cited way of preventing malaria. Three-fourths of the respondents (75.6%) were definitely sure that malaria was the most common disease experienced in their community (Table 3).

Over half of the respondents were definitely sure that pregnant women (61.1%) and children (71.5%) were at risk for malaria. The method of preventing malaria in pregnancy which most respondents definitely knew about was sleeping under long-lasting ITN (63.5%). More than one-third (37.5%) were definitely unaware of IPTp (Table 4).

Less than half of the respondents (31.1%) were definitely convinced that the community had a role to play in preventing malaria in pregnant women and children. The respondents definitely perceived that ensuring that family members sleep under ITN (53.1%) and sustaining efforts of projects to prevent malaria in pregnancy even after project ends (49.4%) as community roles in preventing malaria in pregnancy (Table 5).

Majority of the participants (60.6%) strongly agreed that community-directed distribution of IPTp-SP will improve prevention of malaria in pregnancy. About half of the respondents strongly agreed that community-directed distribution of SP was acceptable (49.2%) and were willing to support the project (50.8%). Majority (74.4%) of the pregnant respondents preferred community administration to facility administration of IPTp (Table 6).

**Figure 1:**
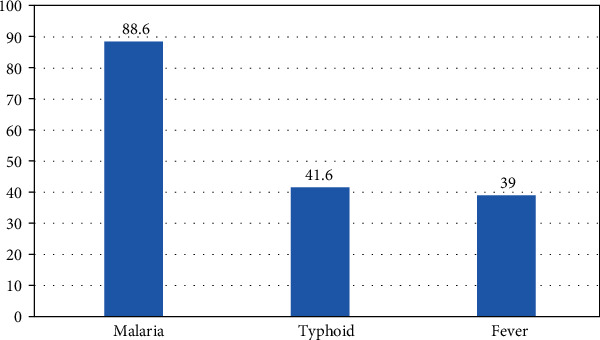
Three major health conditions in the community identified by community members in Ebonyi, Nigeria.

Malaria (88.6%), typhoid (41.6%), and fever (39.0%) were the three major health conditions in the community identified by the participants (Figure 1).

### 3.2. Qualitative Findings

One of the FGDs consisted of seven men and five women aged 24-65 years while the second FGD comprised of ten pregnant and nursing mothers aged 18-40 years.

### 3.3. Major (Prevalent) Health Problems in the Community

The major health challenges mentioned by the community members included malaria, fever, arthritis, peptic ulcer, convulsions in children, and eye problems in the elderly.

### 3.4. Knowledge of Malaria

#### 3.4.1. Misconceptions about Malaria Transmission

All the participants were aware of malaria and gave the names for malaria in the local parlance. Malaria was known as *isi-owuwa*, *aru oku*, and *iba* in the local dialect. On more probing regarding the cause of malaria, the participants gave varied opinions ranging from mosquito bites, poor personal and environmental hygienic conditions, work stress, and eating stale food.

Some of the verbatim responses are below:

“You can contact malaria through mosquito bites. Secondly drinking unclean water and through dirty hands.” (Younger male).

“Eating food that is not warmed very well is another way of getting malaria because cold food gives malaria.”(Older male).

#### 3.4.2. Symptoms of Malaria

The participants readily mentioned the various symptoms of malaria, and this included headache, fever, body pains, excessive sleep, vomiting, and weakness.

#### 3.4.3. Population Vulnerable to Malaria

All the participants identified pregnant women and children to be susceptible to malaria.

#### 3.4.4. Effects of Malaria in Pregnancy

The discussants identified miscarriages, maternal anemia, poor growth of the foetus, and jaundice in the newborn as effects of malaria during pregnancy in addition to other symptoms of malaria which were shared by women who had experienced malaria in pregnancy.

#### 3.4.5. Prevention of Malaria

The participants exhibited good knowledge of malaria prevention through avoidance of stagnant water and always sleeping under a net. One of the discussants highlighted the importance of sleeping under ITN and treatment for pregnant women thus:

“Pregnant woman should always sleep under the net even after delivery but when the person is infected she should be treated” (Female community member).

#### 3.4.6. Intermittent Preventive Therapy for Malaria in Pregnancy

Only a few of the participants were aware of IPTp. Knowledge was based on direct experience via use while pregnant and indirectly through the pregnant wife experience.

This is illustrated by the comment below:

“I know about it. I got to know about it when my wife came for antenatal care. She came back with the drugs and showed it to me that they asked her to take it once. She took the drugs and there was a difference in her body” (Male community member).

### 3.5. Acceptability of Community-Directed Distribution of IPTp

The participants were asked to how acceptable they considered the community-directed distribution of IPT project. Before this, participants were informed that the project would involve monthly distribution of a drug designed to prevent malaria in pregnancy (and typically taken in the health facility) to pregnant women at in the community. All the participants assented that the project was acceptable and were willing to support it. One of the participants felt that community–directed distribution will ease the burden of work on health workers in health facilities. The participants were also of the opinion that this would not discourage antenatal clinic attendance.

“It (community-directed IPTp-SP distribution) is good so that the nurses can have rest.” (Male community member) (Chorus laughter and nodding in agreement following this comment).

“If the drugs are shared the way it's been done before (referring to Mectizan distribution), the villagers will accept it.” (Female community member).

“We know every family in this community and we will inform our people to accept the drugs; after all, we are the ones benefitting from it.”(Male community leader).

“I don't think it will make women stop antenatal care, they will still be coming” (Female community member).

### 3.6. Perceived Potential Challenges of Community Distribution of IPTp

The community members highlighted two main challenges that community distribution of IPTp could encounter. These were reduced uptake due to the fear of side effects and previous rejection of distributed drugs due to spread of false rumors. To address this, the community reinstated the need for active participation and education of community members. Some comments to highlight these are below:

“There was a time a particular drug was distributed, and the news was that when a person uses it he/she will die. Because of that, people stopped collecting drugs from strangers. So the community members should be involved so that when pregnant women see them, they will collect the drugs.” (Male community leader).

“One challenge is that when people take some distributed drugs, the drug will bring out other negative effects and this can cause people to stop taking drugs.” (Female community member).

### 3.7. Potential for Sustainability of Community Distribution of IPTp

Majority of the participants were inclined towards sustaining distribution conditional on availability of the drugs. One of the participants agreed that there was a potential for sustainability of community distribution of IPTp through the use of community contributions to fund procurement of the drugs. The statement below portrays this opinion:

“If the community sees the effectiveness of the drug, they can now decide to contribute together to help bring the drug for those who need it” (Male community member).

## 4. Discussion

This study presents the baseline findings of an intervention study that implement community-directed distribution of intermittent preventive therapy for prevention of malaria in pregnancy. It explored community malaria experiences, knowledge, and perceived roles in malaria prevention in pregnancy. It also assessed the acceptability of community-directed distribution of *sulfadoxine-pyrimethamine* for malaria in pregnancy.

Regarding malarial burden and experiences, a greater proportion of the respondents identified malaria as the most common health condition in the community. The prevalence of fever and diagnosis of malaria in the past one year reported by the participants was also remarkably high. This was also in consonance with the FGD findings whose participants went further to describe the local names for malaria. Although this finding was based on self-reports, it further buttresses the already known burden and endemicity of malaria in Nigeria especially in the rural areas [7]. Other studies among rural communities have also found also that malaria was reported to be the most common disease in their settings [27–30]. Although most respondents who reportedly had malaria were treated with antimalarial drugs, the use of herbal concoctions and unidentified drugs from patent medicine venders calls for concern. Malaria care seeking from traditional healers and unqualified providers is common in the rural areas [20]. Inadequate treatment of malaria could sustain the morbidity and contribute to drug resistance especially with single/incomplete dose drug mixtures [31, 32]. However, the authors did not ascertain whether those who received antimalarial were treated with Artemisinin-based combination therapy as globally and nationally recommended [33, 34].

This study revealed that most of the participants knew the cause of malaria, population groups most susceptible to malaria infection, and the effects of malaria in pregnancy. This was further corroborated by the FGD findings. The fact that malaria is endemic in Nigeria with many community-level control activities including awareness creation in place could account for this high knowledge levels. Also, malaria was highlighted as the most common ailment in the community, and this could also account for the knowledge levels. Some studies have found poor knowledge of the cause of malaria among community members in Nigeria [35].

Apart from ITN use, there was suboptimal knowledge of other methods for prevention of malaria. In contrast, knowledge of malaria prevention measures has been found to be higher in other studies [35]. Sleeping under ITN was the major preventive method cited by the respondents. The use of ITN was also highlighted during the focus group discussions. The prominence of ITN could be because most respondents had ITNs. Insecticide treated mosquito net was the most known and used malaria preventive method in other community-based studies in Nigeria, Kenya, and Mozambique [8, 9, 35]. Wrong methods of prevention such as use of herbal medications for preventing malaria was mentioned by some of the respondents. Additionally, some misconceptions on the etiology of malaria such as excessive sunlight exposure, witchcraft, and food consumption (oily foods, spoilt foods) were identified from the survey and FGDs. Such misconceptions and lacunae have been reported in other studies [36, 37].

Majority of the participants had poor knowledge of IPTp, and this was demonstrated in both the question-based and self-graded assessment of their knowledge of IPTp. Only few of the respondents could identify the commonly used brands of sulfadoxine-pyrimethamine used for IPTp, namely, Fansidar and Amalar. This finding was also supported by the FGD participants; most of whom demonstrated poor knowledge of IPTp except for pregnant women and husbands who knew that their wives had received sulfadoxine-pyrimethamine in the past. Correspondingly, another study in Southeast Nigeria found that community members could not identify Fansidar as the drug for IPTp [27]. Some possible reasons for this inadequate knowledge could be because IPTp is only taken by pregnant women with most educational activities traditionally focused on them in healthcare settings [38–41]. Likewise, studies conducted among community members have found low levels of knowledge of malaria treatment [42]. Although the wider community (men and older women inclusive) may not have been optimally engaged on IPTp in the past, they have been found to play key roles in promoting interventions aimed at reducing malaria in pregnancy [43]. Thus, it is important that community engagement and knowledge translation is integrated into programs to control and/or eliminate malaria in pregnancy. Although no additional information on IPTp was provided, most opined that IPTp would be effective in preventing malaria in pregnancy. This could be because in the study area's context, people may believe that since these drugs were manufactured by scientists and given to pregnant women (who are delicate), it should be effective. As seen from the focus group discussions and other results, the respondents knew that ITNs are protective against malaria in pregnancy. The knowledge may also influence their thinking that such drugs specially made for malaria in pregnancy should be effective as well.

Most participants affirmed that the community had a role to play in malaria prevention in pregnancy. The aspects of community roles explored include referral promoting antenatal care, promoting use of IPTp, ensuring family members sleep under ITN, referral of febrile pregnant women and children to the health facility, and collaborating with and sustaining projects on malaria prevention in pregnancy. Across all these domains, the majority of the respondents had positive perceptions towards community involvement. The felt burden of malaria in the community could have contributed to this positive role perception. Community involvement and ownership is critical to the success of community-level projects [12]. The successful implementation of the community-directed distribution of *sulfadoxine-pyrimethamine* project in the study community was mostly hinged on the positive attitude and cooperation shown by the community members who were actively involved in the project [19].

Social constraints such as acceptability can determine the outcome of community interventions for health problems in Africa [12]. In this study, the majority of the respondents considered the community-directed distribution of *sulfadoxine-pyrimethamine* acceptable and were willing to support the project. Some reasons given for the acceptability of the project by the FGD participants were previous favorable, experience with Mectizan drug distribution for neglected tropical diseases, reduction of workload in the health facility, and the beneficial effects of the project to the community. The FGD participants also expressed willingness to publicize the project in the community in order to promote uptake. Similar studies that evaluated community perception towards community-based programs such as community case management of malaria by community health workers also found positive community attitudes. Reduced queues in the health facilities was cited as the main reason for this disposition [18]. The high levels of acceptability closely match with the postintervention satisfaction levels with the community-directed distribution of IPTp intervention [19].

This study is not without a few limitations. Firstly, the limited number of communities involved in the survey limits the representativeness of the findings. The use of mothers recruited from health facilities could have introduced sampling bias; however, data was also collected from women of child-bearing age/mothers regardless of pregnancy status in the community. The recruitment of mothers accessing ANC/EPI services was to enhance adequate representation and inclusion of mothers who were the primary targets of the community-directed distribution. Also, the findings were based on self-reports which could be prone to social desirability bias. To mitigate confirmation bias during the FGDs, the moderator regularly summarized and clarified that responses were well captured. The moderator was careful to maintain a neutral disposition towards responses by the participants, and the researchers collated and evaluated all data equally. Since a questionnaire and discussion guide was used, question-order bias could have occurred; however, this was mitigated by pretesting, grouping of questions, and the interviewers adopted a random approach to asking the questions. Data was checked and validated and stored in a password-enabled computer only accessible to the primary researcher.

## 5. Conclusions

This study has shown that the community members surveyed recognized malaria as a prevalent disease in the community with significant symptomatic burden. There was good knowledge of malaria etiology and susceptible populations; however, knowledge of malaria prevention in pregnancy notably intermittent preventive therapy for malaria in pregnancy was suboptimal. There was positive perception of roles in malaria prevention in pregnancy and acceptability of community-directed distribution of intermittent preventive therapy for malaria in pregnancy. There was positive perception of roles in malaria prevention in pregnancy and high acceptability of community-directed distribution of IPTp. Community level malaria control programs should utilize a whole-of–community approach to optimally engage and educate the community on malaria prevention in pregnancy as well as explore community distribution approach for IPTp.

## Figures and Tables

**Table 1:** Socio-demographic characteristics of community members in Ebonyi, Nigeria (*n* = 817).

Variable	Frequency	Percent (%)
Age (mean ± SD)	36.08 ± 15.4	
Gender		
Male	603	73.8
Female	214	26.2
Marital status		
Unmarried	160	19.6
Married	616	75.4
Divorced	2	0.2
Separated	5	0.6
Widowed	34	4.2
Educational level		
No formal education	117	14.3
Primary education	233	28.5
Secondary education	401	49.1
Postsecondary education	66	8.1
Religion		
Christian	802	98.2
Traditional religion	12	1.5
Islam	2	0.2
Others^	1	0.1
Employment status		
Unemployed	67	8.2
Paid employment	65	8.0
Self-employment^+^	685	83.8

^Others: Atheist ^+^farmers, traders, artisans.

**Table 2:** Malaria-related characteristics of community members in Ebonyi, Nigeria (*n* = 817).

Variable	Frequency	Percent (%)
Household net possession/type		
Windows with nets	77	9.4
Netted doors and windows	59	7.2
Ordinary mosquito nets	30	3.7
Long-lasting ITN	533	65.2
None	220	26.9
Source of ITN (*n* = 533)		
Health facility	350	65.7
Personal purchase	9	1.7
Friend/family member	17	3.2
Medical outreach	154	28.9
Others	3	0.6
Ever had fever in the past one year		
Definitely yes	548	67.1
Probably yes	43	5.3
Unsure	1	0.1
Probably no	21	2.6
Definitely no	204	25.0
Was this fever diagnosed as malaria (*n* = 591)		
Yes	481	81.4
No	73	12.4
Unsure	37	6.2
Where and how was the malaria diagnosed (*n* = 481)		
Health facility using microscopy	5	1.0
Chemist using symptoms	33	6.7
Health facility using RDT	338	70.3
Health facility using history	10	2.0
Chemist using RDT	14	2.9
By family/friends	3	0.6
Self-diagnosed using usual symptoms	78	15.9
How was the fever treated (*n* = 591)		
Use of antimalarial from a chemist	36	6.1
Drugs mixed form chemist	79	13.4
Use of antimalarial from a health facility	344	58.2
Antibiotics	3	0.5
Paracetamol	9	1.5
Herbal drugs/concoctions	99	16.8
No treatment	21	3.5

ITN: Insecticide-Treated Net; RDT: rapid diagnostic test.

**Table 3:** General knowledge of malaria and intermittent preventive therapy in pregnancy (IPTp) among community members in Ebonyi, Nigeria (*n* = 817).

Variable	Frequency	Percent (%)
Cause of malaria		
Infected mosquito bites	605	74.1
Too much sun	84	10.3
Others	76	9.3
Witchcraft	5	0.6
Oily foods/groundnuts	47	5.7
Is malaria the most common disease experienced in this community?		
Definitely no	20	2.4
Probably no	52	6.4
Unsure	22	2.7
Probably yes	105	12.9
Definitely yes	618	75.6
Ever heard of/aware of IPTp		
Definitely no	306	37.5
Probably no	101	12.4
Unsure	13	1.6
Probably yes	125	15.3
Definitely yes	272	33.3
Knowledge of drug used for IPTp		
Do not know	682	83.5
Fansidar	59	7.2
Chloroquine	20	2.4
Amalar	17	2.1
Phensic	3	0.4
Others	36	4.4
Self-graded knowledge of IPTp		
Grossly inadequate	380	46.5
Inadequate	141	17.3
Fairly adequate	146	17.9
Adequate	62	7.6
Very adequate	88	10.7
Ways of preventing malaria (apart from IPTp)		
Sleeping under treated mosquito nets	444	54.3
Early and complete treatment	165	20.2
Clean environment	324	39.7
Indoor residual spraying	142	17.4
Herbal medicines	219	26.8
Do not know	59	7.2
Others	29	3.5

IPTp: intermittent preventive therapy in pregnancy.

**Table 4:** Knowledge of at-risk groups for malaria and prevention of malaria in pregnancy (*n* = 817).

Variable	Definitely no	Probably no	Unsure	Probably yes	Definitely yes	Mean ± SD
Pregnant women are mostly affected by malaria	46 (5.6)	33 (4.0)	86 (10.5)	153 (18.7)	499 (61.1)	4.26 ± 1.1
Under-five children are mostly affected by malaria	24 (2.9)	15 (1.8)	37 (4.5)	157 (19.2)	584 (71.5)	4.54 ± 0.9
Sickle cell disease patients are mostly affected by malaria	62 (7.6)	56 (6.9)	362 (44.3)	96 (11.8)	241 (29.5)	3.49 ± 1.2
Malaria can cause serious illness and death in pregnant women and children	31 (3.8)	46 (5.6)	57 (7.0)	191 (23.4)	492 (60.2)	4.31 ± 1.1
Pregnant women and their unborn children can be protected from malaria by sleeping under long-lasting ITN	41 (5.0)	17 (2.1)	23 (2.8)	217 (26.6)	519 (63.5)	4.41 ± 1.0
Pregnant women and their unborn children can be protected from malaria by multiple ingestion of 3 tablets of specified antimalarial while pregnant	14 (1.7)	30 (3.7)	109 (13.3)	276 (33.8)	388 (47.5)	4.22 ± 0.9
Pregnant women and their unborn children can be protected from malaria by early diagnosis and prompt treatment with antimalarial	10 (1.2)	7 (0.9)	73 (8.9)	230 (28.2)	497 (60.8)	4.47 ± 0.8

**Table 5:** Perceived community roles in prevention of malaria in pregnancy and childhood among community members in Ebonyi, Nigeria (*n* = 817).

Variable	Definitely no	Probably no	Unsure	Probably yes	Definitely yes	Mean ± SD
The community has a role to play in preventing malaria in her pregnant women and children	32 (3.9)	58 (7.1)	108 (13.2)	365 (44.7)	254 (31.1)	3.92 ± 1.0
Community can play a role in prevention of malaria in pregnancy by promoting ANC for pregnant women	36 (4.4)	30 (3.7)	77 (9.4)	277 (33.9)	397 (48.6)	4.19 ± 1.0
Community can play a role in malaria prevention in pregnancy by promoting use of IPTp by pregnant women	20 (2.4)	31 (3.8)	107 (13.1)	286 (35.0)	373 (45.7)	4.18 ± 0.9
Community can play a role in malaria prevention in pregnancy by ensuring family members sleep under ITN	22 (2.7)	21 (2.6)	74 (9.1)	266 (32.6)	434 (53.1)	4.31 ± 0.9
Community can play role in malaria prevention in pregnancy by referring pregnant women and children with fever to the health facility	5 (0.6)	26 (3.2)	83 (10.2)	375 (45.9)	328 (40.1)	4.22 ± 0.8
Community should collaborate with partners/projects that promote prevention of malaria in pregnancy	7 (0.9)	22 (2.7)	95 (11.6)	375 (45.9)	318 (38.9)	4.19 ± 0.8
Community should sustain efforts of projects to prevent malaria in pregnancy even after the end of the project	13 (1.6)	14 (1.7)	105 (12.9)	281 (34.4)	404 (49.4)	4.28 ± 0.9
Health projects conducted in the community should carry the community members along	9 (1.1)	15 (1.8)	84 (10.3)	351 (43.0)	358 (43.8)	4.27 ± 0.8

**Table 6:** Acceptability of community-directed distribution of IPTp-SP among community members in Ebonyi, Nigeria.

Parameter assessed	Strongly disagree	Disagree	Neutral	Agree	Strongly agree	Mean ± SD
Community-directed distribution of SP will improve prevention of malaria in pregnancy	14 (1.7)	26 (3.2)	67 (8.2)	215 (26.3)	485 (60.6)	4.41 ± 0.9
Community-directed distribution of SP is good and acceptable to me	23 (2.8)	58 (7.1)	97 (11.9)	237 (29.0)	402 (49.2)	4.15 ± 1.1
I am willing to support community-directed distribution of SP project	25 (3.1)	37 (4.5)	55 (6.7)	285 (34.9)	415 (50.8)	4.26 ± 0.9
Acceptability of community-directed distribution of IPTp-SP among pregnant women only (*n* = 242)						
I would rather take IPT in the community than in the health facility	16 (6.6)	32 (13.2)	14 (5,8)	52 (21.5)	128 (52.9)	4.01 ± 1.3

## Data Availability

The datasets used and/or analyzed during the current study are available from the corresponding author on reasonable request (ijeomaninadr@gmail.com).

## Abbreviations

FGDs: Focus group discussions
IPTp: Intermittent preventive treatment in pregnancy
IPTp-SP: Intermittent preventive treatment with sulfadoxine-pyrimethamine
ITN: Insecticide-Treated Net
LBW: Low birth weight
MIP: Malaria in pregnancy
PHC: Primary health center
